# Supplementary material for: Comparative genomics provides new insights into the diversity, physiology, and sexuality of the only industrially exploited tremellomycete: Phaffia rhodozyma
Source: BMC Genomics. 2016 Nov 9;17:901. doi: 10.1186/s12864-016-3244-7 (PMC5103461; doi:10.1186/s12864-016-3244-7)
Supplement: Additional file 6: — List of orphan genes with links to PFAM (related to Additional file 1: Table S1). (ZIP 1428 kb) [file 12864_2016_3244_MOESM6_ESM.zip › PFAM_UNIQUE/G04938_P.html]

#                                                                            --- full sequence --- -------------- this domain -------------   hmm coord   ali coord   env coord
  
# target name        accession   tlen query name           accession   qlen   E-value  score  bias   #  of  c-Evalue  i-Evalue  score  bias  from    to  from    to  from    to  acc description of target
  
#------------------- ---------- ----- -------------------- ---------- ----- --------- ------ ----- --- --- --------- --------- ------ ----- ----- ----- ----- ----- ----- ----- ---- ---------------------
  
G04938\_P             -            220 ATPase\_gene1         PF09527.5     55     0.068   11.7   0.3   1   1   7.4e-05      0.11   11.0   0.3     2    29   124   151   123   154 0.89 - Putative F0F1-ATPase subunit (ATPase\_gene1)  
G04938\_P             -            220 ETRAMP               PF09716.5     84      0.11   11.1   2.0   1   2   0.00027      0.81    8.4   1.0    54    80   125   149    41   153 0.66 - Malarial early transcribed membrane protein (ETRAMP)  
G04938\_P             -            220 ETRAMP               PF09716.5     84      0.11   11.1   2.0   2   2     0.037   1.1e+02    1.5   0.0    36    64   181   212   154   215 0.63 - Malarial early transcribed membrane protein (ETRAMP)  
G04938\_P             -            220 PHO4                 PF01384.15   326     0.008   13.5   0.1   1   1   9.9e-06    0.0098   13.2   0.1    83   157    86   159    83   196 0.71 - Phosphate transporter family  
G04938\_P             -            220 PMP1\_2               PF08114.6     43     0.037   12.2   1.8   1   1   1.1e-05     0.067   11.4   1.8    10    37   127   154   122   156 0.92 - ATPase proteolipid family  
G04938\_P             -            220 TMEM154              PF15102.1    146     0.016   13.6   0.1   1   2   6.9e-05     0.041   12.3   0.0    36   119    99   188    84   194 0.65 - TMEM154 protein family  
G04938\_P             -            220 TMEM154              PF15102.1    146     0.016   13.6   0.1   2   2       1.5   9.3e+02   -1.9   0.1    12    32   195   214   189   217 0.62 - TMEM154 protein family  
G04938\_P             -            220 Yip1                 PF04893.12   172     0.063   11.5   0.1   1   1   0.00024      0.09   10.9   0.1    38    86   100   148    66   154 0.85 - Yip1 domain
